# Pipelined Key Switching Accelerator Architecture for CKKS-Based Fully Homomorphic Encryption

**DOI:** 10.3390/s23104594

**Published:** 2023-05-09

**Authors:** Phap Ngoc Duong, Hanho Lee

**Affiliations:** Department of Information and Communication Engineering, Inha University, Incheon 22212, Republic of Korea

**Keywords:** fully homomorphic encryption (FHE), key switching, homomorphic multiplication, Cheon–Kim–Kim–Song (CKKS), number theoretic transform (NTT)

## Abstract

The increasing ubiquity of big data and cloud-based computing has led to increased concerns regarding the privacy and security of user data. In response, fully homomorphic encryption (FHE) was developed to address this issue by enabling arbitrary computation on encrypted data without decryption. However, the high computational costs of homomorphic evaluations restrict the practical application of FHE schemes. To tackle these computational and memory challenges, a variety of optimization approaches and acceleration efforts are actively being pursued. This paper introduces the KeySwitch module, a highly efficient and extensively pipelined hardware architecture designed to accelerate the costly key switching operation in homomorphic computations. Built on top of an area-efficient number-theoretic transform design, the KeySwitch module exploited the inherent parallelism of key switching operation and incorporated three main optimizations: fine-grained pipelining, on-chip resource usage, and high-throughput implementation. An evaluation on the Xilinx U250 FPGA platform demonstrated a 1.6× improvement in data throughput compared to previous work with more efficient hardware resource utilization. This work contributes to the development of advanced hardware accelerators for privacy-preserving computations and promoting the adoption of FHE in practical applications with enhanced efficiency.

## 1. Introduction

**Figure 1:**
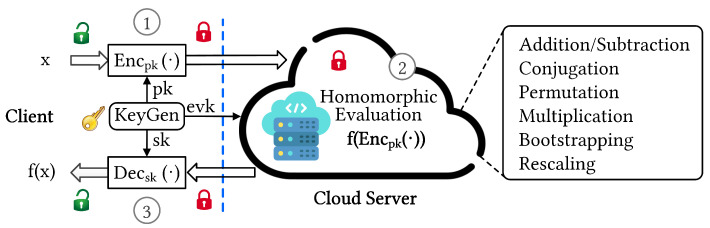
Overall FHE-based cryptosystem with main operations: (1) encryption, (2) homomorphic evaluation, and (3) decryption.

With the explosion of the Internet-of-Things-based data and the widespread use of machine learning (ML) as a cloud-based service, securing private user data during ML inferences has become a pressing concern for cloud-service providers. Fully homomorphic encryption (FHE) is a promising solution for preserving sensitive information in cloud computing because it provides strong defense mechanisms and enables the direct computation on encrypted data (ciphertext) while preserving confidentiality [1,2]. However, the requirement for high degrees of security leads to complex parameter settings, resulting in expensive computation on large ciphertext, which limits the practical realization of FHE-based applications. Cloud-side analytics can be resource-intensive and time-consuming, making it necessary to develop cryptographic accelerators to facilitate the deployment of real-world applications. Cryptographic accelerators are designed to reduce the computational overhead of homomorphic functions, thus enabling faster and more efficient computation on encrypted data. The development of such accelerators is crucial to unlock the full potential of FHE-based solutions, make it more accessible to a wider range of users and supporting the secure processing of sensitive data in real-world settings. Figure 1 illustrates an end-to-end FHE-based cryptosystem with primary homomorphic operations performed in the cloud server.

FHE cryptographic protocols typically involve integer- and lattice-based schemes. The most efficient lattice-based schemes rely on the ring learning with errors (RLWE) problem, which provides strong security guarantees and the desired performance [3]. In RLWE-based FHE protocols, the input messages are encrypted by adding noise, and the generated ciphertexts are composed of two polynomial rings. The growth of noise through homomorphic computations limits the circuit depth, and the selection of FHE parameters must balance the security requirements with computational complexity [4]. Parameter selection primarily involves polynomial degree *N*, and modulo integer *Q* with at least 128-bit security is typically required to guard against unpredictable attacks [5]. To support multiplicative depth, *N* increases proportionally. High-circuit-depth FHE schemes inevitably have the drawback of large ciphertexts, which leads to expensive computations, high-bandwidth data movement, and large storage-space requirements.

**Figure 2:**
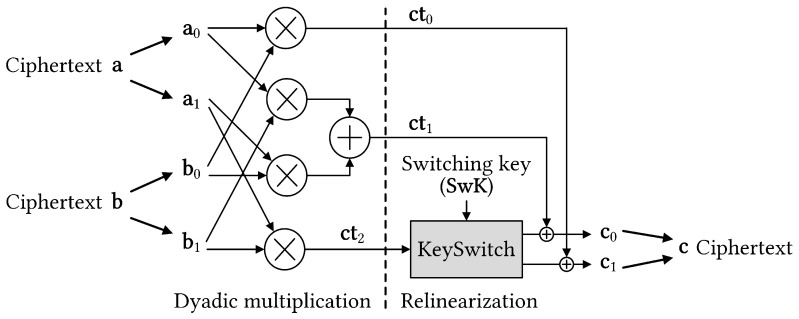
Ciphertext multiplication involving the relinearization step (that is, key switching operation).

Primary homomorphic operations involve addition, multiplication, and permutation of ciphertexts. Homomorphic multiplication between ciphertexts is often computationally expensive because of the convolution of polynomial coefficients. Figure 2 shows a general diagram of the multiplication between two ciphertexts that dominates homomorphic operations. Initially, ciphertext consists of two component polynomials. The ciphertext multiplication results in a tuple of polynomials, making further computation challenging. Thus, an operation is required to revert the ciphertext to its original form. An expensive operation known as key switching is required to relinearize the ciphertext. However, key switching is computationally intensive with number theoretic transform (NTT) and inverse NTT (INTT) operations being dominant. Therefore, developing key switching hardware accelerators is significant for speeding up homomorphic multiplication and realizing FHE-based applications.

### 1.1. Related Works

While FHE holds potential, its primary limitation is inefficiency, which stems from two factors: complex polynomial operations and time-consuming ciphertext management. To tackle the computational and memory demands of homomorphic functions, various optimization and acceleration efforts are underway. Table 1 presents FHE accelerators, highlighting the hardware utilized and features of the accelerators. Initially, FHE acceleration depended on general hardware features. However, CPUs lack the capacity to effectively harness FHE’s inherent parallelism [6]. GPU-based implementations tap into this parallelism, but GPU’s extensive floating-point units remain underused as FHE tasks mainly involve integer operations [7,8,9]. Furthermore, neither CPUs nor GPUs offer sufficient main memory bandwidth to cope with FHE workload’s data-intensive nature.

To enhance FHE scheme performance, researchers have been exploring custom hardware accelerators using ASIC and FPGA technologies. ASIC solutions [10,11,12,13] show promise, as they surpass CPU/GPU implementations and bridge the performance gap between plaintext and ciphertext computations. However, to accommodate large on-chip memory, expensive advanced technology nodes such as 7 nm or 12 nm are required for ASIC implementations. Furthermore, designing and fabricating these ASIC proposals demand significant engineering time and high non-recurring costs. Since FHE algorithms are not standardized and continue to evolve, any changes would necessitate major ASIC redesign efforts. Conversely, FPGA solutions are more cost-effective than ASICs, offer rapid prototyping and design updates, and are better equipped to adapt to future FHE algorithm modifications.

Several studies have proposed FPGA-accelerated architecture designs for FHE [14,15,16,17,18,19]. Notably, Riazi et al. introduced HEAX, a hardware architecture that accelerates CKKS-based HE on Intel FPGA platforms and supports low parameter sets [14]. However, the architecture faces high input/output and memory interface bandwidths, as well as costly internal memory, making it difficult to place and route multiple cores on the target FPGA platform. Han et al. proposed coxHE, an FPGA acceleration framework for FHE kernels using the high-level synthesis (HLS) design flow [16]. Targeting key switching operations, coxHE examined data dependence to minimize interdependence between data, maximizing parallel computation and algorithm acceleration. Mert et al. proposed Medha, a programmable instruction-set architecture that accelerates cloud-side RNS-CKKS operations [17]. Medha featured seven residue polynomial arithmetic units (RPAU), memory-conservative design, and support for multiple parameter sets using a single hardware accelerator with a divide-and-conquer technique. However, these three FPGA-based implementations only support small parameter sets, insufficient for bootstrapping. Recently, Yang et al. proposed Poseidon, an FPGA-based FHE accelerator supporting bootstrapping on the modern Xilinx U280 FPGA [18]. Poseidon employed several optimization techniques to enhance resource efficiency. Similarly, Agrawal et al. presented FAB, an FPGA-accelerated design that balances memory and computing consumption for large homomorphic parameter bootstrapping [19]. FAB accelerates CKKS bootstrapping using a carefully designed datapath for key switching, taking full advantage of on-chip 43 MB on-chip storage. However, the design’s extensive parallelism consumes numerous logic elements, especially with larger parameter sets. Additionally, inefficient scheduling can result in redundant resource consumption and complex workflow synchronization, leading to suboptimal performance. In this work, we adopt a pipelined KeySwitch design to simplify scheduling and target high-throughput implementation. Our design method leverages FPGA fabric’s programmable logic elements and enhances on-chip memory utilization.

### 1.2. Our Main Contributions

This study presents a comprehensive hardware architecture for the KeySwitch accelerator design, which operates in a highly pipelined manner to speed up CKKS-based FHE schemes. Built on compact NTT and INTT engines [20], the KeySwitch module efficiently employs on-chip resources. Importantly, our design approach significantly reduces internal memory consumption, allowing on-chip memory to hold temporary data. The design executes subfunctions concurrently in a pipelined and parallel manner to boost throughput. We demonstrate an example design supporting a three-level parameter set. The proposed KeySwitch module was evaluated on the Xilinx UltraScale+ XCU250 FPGA platform, and we provide an in-depth discussion of the design methodology and area breakdown for better understanding of key operations. Compared to the most related study, our KeySwitch module achieves a 1.6x higher throughput rate and superior hardware efficiency.

The remainder of this paper is organized as follows: Section 2 provides an overview of the underlying operations of RLWE-based HE schemes. Section 3 describes the key switching algorithm in detail, and Section 4 presents the design of our KeySwitch module. Section 5 presents the experimental results, compares our approach with related works, and discusses our findings. Finally, Section 6 concludes the study.

## 2. Background

CKKS-based HE schemes have been extensively studied to perform meaningful computations on encrypted data of real and complex numbers. In the encrypted data domain, the ciphertext often consists of two *N*-degree polynomials, and each coefficient is an integer modulo *Q*. Therefore, the underlying homomorphic operations in RLWE-based HE schemes share similarities, enabling the development of a single hardware accelerator that can support multiple HE instances. Our study primarily focuses on accelerating CKKS-based homomorphic encryption; however, the operations described at the ciphertext level have a broad applicability to almost all lattice-based homomorphic encryption schemes.

### 2.1. Residue Number System

The Chinese remainder theorem (CRT) enables a polynomial in $R_{Q}$ to be represented as an RNS decomposition with smaller pairwise coprimes such that $Q=\prod_{i=0}^{L}q_{i}$ [21]. This enables polynomial a in $R_{Q}$ to be represented in RNS channels as a set of polynomial components. For instance, considering an RNS representation with three pairwise co-prime moduli $q_{0},\;q_{1},\;q_{2}$, the polynomial a can be represented as a set of three polynomials: $a\equiv \left(a_{0},\;a_{1},\;a_{2}\right)\;\mathrm{mod}\;\left(q_{0},\;q_{1},\;q_{2}\right)$, where each $a_{i}$ is a polynomial in $Rq_{i}$. This technique can significantly reduce the magnitude of coefficients and improve the performance of arithmetic operations in HE.

(1) $$a=\left({\left[a\right]}_{q_{0}},\ldots ,{\left[a\right]}_{q_{i}}\right)\in \prod_{i=0}^{L}R_{q_{i}}$$

We denote the polynomial component in a ring field $R_{q_{i}}=Z_{q_{i}}$/$\left(X^{N}+1\right)$ as follows:

(2) $${\left[a\right]}_{q_{i}}=a_{0}+a_{1}X+\ldots +a_{N-1}X^{N-1}\in R_{q_{i}}$$

Thus, arithmetic operations on large integer coefficients can be performed for each smaller modulus without any loss of precision.

### 2.2. Gadget Decomposition

Let *q* be the modulus and $g=\left(g_{0},g_{1},\ldots ,g_{d-1}\right)\in Z^{d}$ be a gadget vector. A gadget decomposition [22], denoted by $g^{-1}:Z_{q}\to Z^{d}$, maps an integer $a\in Z_{q}$ into a vector $\bar{a}=g^{-1}\left(a\right)\in Z_{q}^{d}$ and $\langle g^{-1}\left(a\right),g\rangle =a$ (mod *q*). By extending the domain of the gadget decomposition $g^{-1}$ from $Z_{q}$ to $R_{q}$, we can apply it to a polynomial $a=\sum_{i\in [N]}a_{i}\cdot X^{i}$ in $R_{q}$ by mapping each coefficient $a_{i}$ to a vector $g^{-1}\left(a_{i}\right)\in Z_{q}^{d}$ and then replacing $a_{i}$ with $g^{-1}\left(a_{i}\right)\cdot X^{i}$ in the polynomial expression ($g^{-1}:R_{q}\to R^{d}$ with $a=\sum_{i\in [N]}a_{i}\cdot X^{i}\to \sum_{i\in [N]}g^{-1}\left(a_{i}\right)\cdot X^{i}$). This extension was proposed by [23].

RNS representation can also be integrated with prime decomposition, as exemplified in [24]. An element $a\in R_{Q}$ can be represented in RNS form as ${\left({\left[a\right]}_{q_{i}}\right)}_{0\leq i\leq l}\in \prod_{i=0}^{l}R_{q_{i}}$. The inverse mapping, which allows the retrieval of the original element a from its RNS form, is defined by the formula $a=\sum_{i=0}^{l}a_{i}\cdot g_{i}\cdot {\left[g_{i}^{-1}\right]}_{q_{i}}$ (mod *Q*), where $g_{i}=\frac{Q}{q_{i}}$ [14].

### 2.3. Key Generation

The client begins by generating a secret key sk, which is a polynomial in $R_{Q}$. Then, they generate a uniformly random polynomial r from $U(R_{Q})$ and an error or noise polynomial e from a distribution χ. The corresponding public key is generated as $\mathrm{pk}=\left(b,r\right)\in R_{Q}^{2}$, where b is obtained by taking the inner product of r and a fixed vector s, and adding the error polynomial e, that is, b=〈r,s〉+e.

Let ${\mathrm{sk}}^{\prime }$ be a different key: We sample $D_{1}\leftarrow U\left(R_{Q}^{L}\right)$ and $e\leftarrow \chi^{L}$. Using the gadget vector g, we compute $D_{0}=-{\mathrm{sk}}^{\prime }\cdot D_{1}+\mathrm{sk}\cdot g+e$ (mod *Q*) and return a switching key (**SwK**) as $\mathrm{SwK}=(D_{0,j}|D_{1,j})$, in which $D_{j}$ is a vector of polynomials $d_{i}\in \prod_{i=0}^{l}q_{i}$ [23].

### 2.4. Encryption and Decryption

CKKS encodes a vector of maximal N/2 real values into a plaintext polynomial m of *N* coefficients, modulo *q*. Using the generated public key pk, the client encrypts an input message and produces a noisy ciphertext $\mathrm{ct}=\left(c_{0},c_{1}\right)\in R_{Q}^{2}$ as follows:

(3) $$c_{0}=r_{1}\cdot r+e_{0};c_{1}=r_{1}\cdot b+e_{1}+m$$

where $r_{1}$ is another uniformly random vector and $e_{0}$ and $e_{1}$ are other noise vectors. After homomorphic computations on ciphertexts, the client obtains the results in the encrypted form ${\mathrm{ct}}^{\prime }=\left(c_{0}^{\prime },c_{1}^{\prime }\right)$ and uses the secret key to recover the desired information. Decryption is performed using $m^{\prime }=c_{1}^{\prime }-c_{0}^{\prime }\cdot \mathrm{sk}\approx m+e^{\prime }$ with a small error.

### 2.5. Homomorphic Operations

Homomorphic addition: Taking ciphertexts $a=(a_{0},a_{1})$ and $b=(b_{0},b_{1})$ for example, their homomorphic addition is computed by coefficient-wise adding their co-pair of RNS-element polynomials:

(4) $${\mathrm{ct}}_{add}=a+b=\left(a_{0}+b_{0},a_{1}+b_{1}\right)$$

Homomorphic multiplication: For ciphertexts $a=(a_{0},a_{1})$ and $b=(b_{0},b_{1})$, their homomorphic multiplication is performed by multiplications between their RNS elements:

(5) $${\mathrm{ct}}_{mult}=a\cdot b=\left(a_{0}\cdot b_{0},a_{0}\cdot b_{1}+a_{1}\cdot b_{0},a_{1}\cdot b_{1}\right)$$

This dyadic multiplication produces a special ciphertext of $a_{1}\cdot b_{1}$ for a different secret key (that is, ${\mathrm{sk}}^{2}$). Subsequently, key switching is performed to relinearize the quadratic form of homomorphic multiplication results and obtain a linear ciphertext of the original form.

Key switching: RLWE ciphertexts can be transformed from one secret key to another using key switching computation with **SwK**. This method enables the transformation of a ciphertext decryptable by sk into a new ciphertext under a different secret key ${\mathrm{sk}}^{\prime }$ with an additional error $e_{KS}$. The **SwK** is considered a *d* encryption of $\mathrm{sk}\cdot g_{i}$ under different secret keys ${\mathrm{sk}}^{\prime }$, that is, $\mathrm{SwK}\cdot (1,{\mathrm{sk}}^{\prime })\approx \mathrm{sk}\cdot g$ (mod *Q*) [23].

- Key switching (ct,SwK) return ${\mathrm{ct}}^{\prime }=\left(c_{0},0\right)+g^{-1}\left(c_{1}\right)\cdot \mathrm{SwK}$ (mod *Q*) where $\mathrm{ct}=(c_{0},c_{1})$, $\mathrm{SwK}=(D_{0}|D_{1})$. In detail:
  ${\mathrm{ct}}^{\prime }=\left(c_{0},0\right)+g^{-1}\left(c_{1}\right)\cdot \mathrm{SwK}$
   $=\left(c_{0},0\right)+g^{-1}\left(c_{1}\right)\cdot \left(D_{0},D_{1}\right)$
   $=\left(\left(c_{0}+g^{-1}\left(c_{1}\right)\cdot D_{0}\right),\left(g^{-1}\left(c_{1}\right)\cdot D_{1}\right)\right)=\left(c_{0}^{\prime },c_{1}^{\prime }\right)$
  → $m^{\prime }=c_{0}^{\prime }+c_{1}^{\prime }\cdot {\mathrm{sk}}^{\prime }$
     $=c_{0}+g^{-1}\left(c_{1}\right)\cdot D_{0}+g^{-1}\left(c_{1}\right)\cdot D_{1}\cdot {\mathrm{sk}}^{\prime }$
     $=c_{0}+g^{-1}\left(c_{1}\right)\cdot \left(D_{0}+{\mathrm{sk}}^{\prime }\cdot D_{1}\right)$
     $=c_{0}+g^{-1}\left(c_{1}\right)\left(\mathrm{sk}\cdot g+e\right)$
     $=\langle \mathrm{ct},\left(1,\mathrm{sk}\right)\rangle +e_{KS}$, where $e_{KS}=\langle g^{-1}\left(c_{1}\right),e\rangle$.
  → $m^{\prime }=m+e_{KS}$.

## 3. Key Switching Algorithm

Algorithm 1 provides a detailed description of the homomorphic multiplication with a key switching operation, which is a crucial building block of the SEAL HE library [6]. One remarkable feature of homomorphic multiplication is that NTT is a linear transformation, and optimized HE implementations typically store polynomials in the NTT form across operations instead of their coefficient form. Therefore, the first phase of homomorphic multiplication involves dyadic multiplication. However, the use of the Karatsuba algorithm, a fast multiplication technique, can reduce the total number of coefficient-wise multiplications from four to three. Dyadic multiplication produces a tuple of polynomials ($ct_{0,i}$, $ct_{1,i}$, $ct_{2,i}$), where $ct_{2,i}$ is a special ciphertext that encrypts the square of the secret key; that is, ($1,s,s^{2}$). To recombine the homomorphic products and obtain a linear ciphertext in the form (1,s), key switching is required to make $ct_{2,i}$ decryptable with the original secret key. The homomorphic multiplication is computed using the following equation, which involves key switching using **SwK**:

(6) $${\mathrm{ct}}_{mult}=\left({\mathrm{ct}}_{0},{\mathrm{ct}}_{1}\right)+q_{sp}^{-1}\left({\mathrm{ct}}_{2}\cdot \mathrm{SwK}\right)$$

Key switching is a computationally intensive operation that typically dominates the cost of homomorphic multiplication. The key switching operation requires two inputs: the polynomial component $ct_{2,i}$ and key switching key matrix **SwK**. The polynomial component $ct_{2,i}$ is represented in RNS form as (l+1) residue polynomials, whereas the key switching key matrix SwK=(D0,j|D1,j) is a tensor of (l+1) matrices of (L+2) residue polynomials. RNS decomposition was used to enable fast key switching with a highly parallel and pipelined implementation.

Algorithm 1 shows that key switching involves *l* INTT and $l^{2}$ NTT operations for increasing the modulus, and two INTTs and two *l* NTTs for modulus switching. Thus, key switching dominates the homomorphic multiplication process in terms of the computational cost. However, at *l*-depth level, the main costs are memory expense and data movement. To illustrate the efficient utilization of the on-chip resources on the FPGA platform, we used a parameter set of five modulo primes as a running example. The implementation results indicate that the proposed approach maximizes the utilization of hardware resources.
**Algorithm 1** Homomorphic multiplication algorithm with a key switching operation [6]**Input:** $a=(a_{0},a_{1})$ and $b=\left(b_{0},b_{1}\right)\in {\left(\prod_{i=0}^{l}q_{i}\right)}^{2}$,  $\mathrm{SwK}=\left(D_{0,j}|D_{1,j}\right)\in {\left(q_{sp}\prod_{j=0}^{L}q_{j}\right)}^{2}$  where $D_{j}=d_{i}\in \prod_{i=0}^{l}q_{i}$**Output:** $c=\left(c_{0},c_{1}\right)\in {\left(\prod_{i=0}^{l}q_{i}\right)}^{2}$
 1:/* Dyadic multiplication */ 2:**for** i=0 to *l* **do** 3:  $ct_{0,i}=a_{0,i}⊙b_{0,i}$ 4:  $ct_{1,i}=a_{0,i}⊙b_{1,i}+a_{1,i}⊙b_{0,i}$ 5:  $ct_{2,i}=a_{1,i}⊙b_{1,i}$ 6:**end for** 7:/* Key switching */ 8:**for** i=0 to *l***do**                    ▹ Modulus raising 9:  $\tilde{a}\leftarrow$ INTT${}_{q_{i}}\left(ct_{2,i}\right)$ 10:  **for** j=0 to *l* **do** 11:    **if** i≠j **then** 12:      $\tilde{b}\leftarrow$ Mod$\left(\tilde{a},q_{i}\right)$ 13:      $\bar{b}\leftarrow$ NTT${}_{q_{j}}\left(\tilde{b}\right)$ 14:    **else** 15:      $\bar{b}\leftarrow ct_{2,i}$ 16:    **end if** 17:    ${\bar{c}}_{0,j}\leftarrow {\bar{c}}_{0,j}+\bar{b}⊙d_{0,i,j}$ (mod $q_{j}$) 18:    ${\bar{c}}_{1,j}\leftarrow {\bar{c}}_{1,j}+\bar{b}⊙d_{1,i,j}$ (mod $q_{j}$) 19:  **end for** 20:  $\tilde{b}\leftarrow$ Mod$\left(\tilde{a},q_{sp}\right)$ 21:  $\bar{b}\leftarrow$ NTT${}_{q_{sp}}\left(\tilde{b}\right)$ 22:  ${\bar{c}}_{0,l+1}\leftarrow {\bar{c}}_{0,l+1}+\bar{b}⊙d_{0,i,L+1}$ (mod $q_{sp}$) 23:  ${\bar{c}}_{1,l+1}\leftarrow {\bar{c}}_{1,l+1}+\bar{b}⊙d_{1,i,L+1}$ (mod $q_{sp}$) 24:**end for** 25:**for** k=0 to 1 **do**                  ▹ Modulus switching 26:  $\tilde{r}\leftarrow$ INTT${}_{q_{sp}}\left({\bar{c}}_{k,l+1}\right)$ 27:  **for** i=0 to *l* **do** 28:    r← Mod$\left(\tilde{r},q_{i}\right)$ 29:    $\bar{r}\leftarrow$ NTT${}_{q_{i}}\left(r\right)$ 30:    $c_{k,i}^{\prime }\leftarrow {\bar{c}}_{k,i}-\bar{r}$ (mod $q_{i}$) 31:    $c_{k,i}\leftarrow {\left[q_{sp}^{-1}\right]}_{q_{i}}\cdot c_{k,i}^{\prime }+ct_{k,i}$ (mod $q_{i}$) 32:  **end for** 33:**end for** 34:**return** $c=(c_{0},c_{1})$

## 4. KeySwitch Hardware Architecture

**Figure 3:**
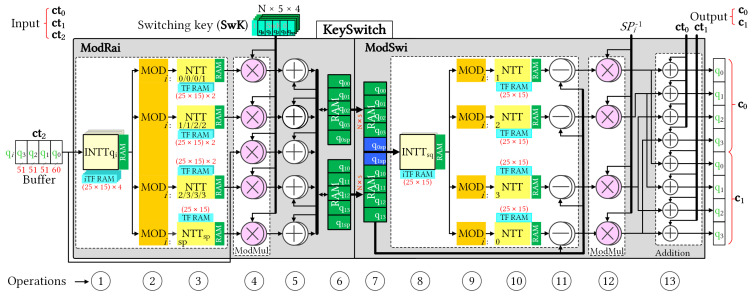
Block-level diagram of the KeySwitch hardware architecture. The components of **ct**${}_{2}$ are stored in Buffer and fed to KeySwitch module in turn. TF and iTF RAM units store bases for associated moduli with 25 × 15 constants for each. In each operation of pipelined stages (i.e., 13 stages of corresponding functions in KeySwitch structure) element units operate in parallel.

Figure 3 illustrates the pipelined architecture of the KeySwitch module with an initial depth of L=3. The KeySwitch module consumes the third component of the dyadic multiplication result and generates relinearized ciphertext. The KeySwitch design was divided in two functional modules with a pipelined connection: ModRai and ModSwi. Two modules have similar structures, and we numbered the sequential operations for clarity. The numbering makes it easier to track the description of their operations.

Key switching operation is computationally intensive, with NTT and INTT operations being dominant. In an FHE setting, ciphertext polynomials are represented in the NTT form by default to reduce the number of NTT/INTT conversions. However, this format is not compatible with the rescaling operation that occurs during moduli switching. Therefore, the key switching process involves performing NTT and INTT operations before and after rescaling, respectively. Consequently, the primary computational costs associated with key switching are for the NTT and INTT operations. Conventionally, the NTT and INTT units consume a large amount of internal memory to store precomputed TFs. In this study, the proposed KeySwitch module employs in-place NTT and INTT hardware designs that aim to reduce the on-chip memory usage [20]. In particular, each NTT and INTT unit stores several TF bases of the associated modulus and utilizes built-in twiddle factor generator (TFG) to twiddle all other factors. Based on the design method of [20] and the exploration of the key switching execution, we designed different NTT modules for associated moduli through pipeline stages. By adopting this approach, the proposed KeySwitch module utilizes hardware resources more efficiently.

**Figure 4:**
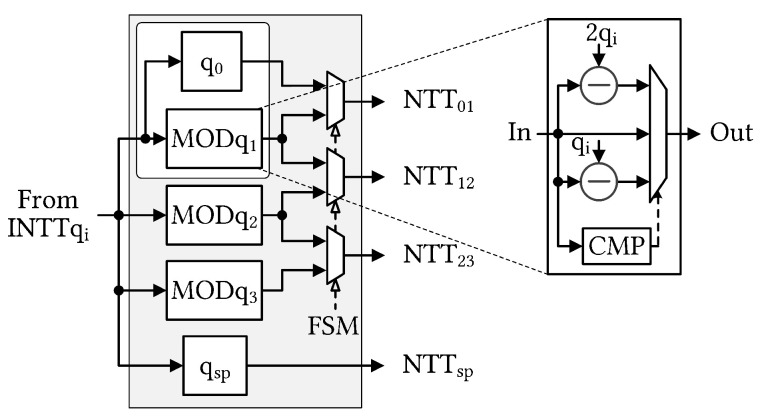
Detailed modular reduction operation (MOD) in op ②. Because $q_{0}$ and $q_{sp}$ are the largest moduli ($q_{sp}$ > $q_{0}$), their MOD operations are eliminated. The CMP unit compares the input with $2q_{i}$ and $q_{i}$.

**Figure 5:**
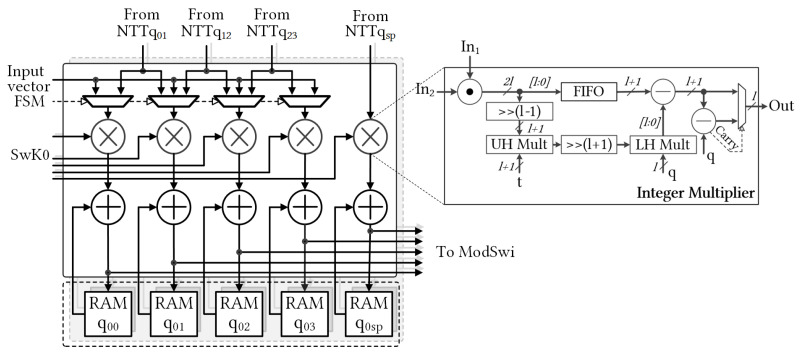
Detailed multiply-accumulate operation (MAR) of ModMul ④, addition ⑤, and random-access memory (RAM) ⑥ in the ModRai module. The ModMul design was presented in [20].

In the ModRai module, the first INTT operation transforms a sequence of (l+1) input polynomials into the associated modulus (op ①). The next stage involves performing MOD operations on the previous INTT results for the (l+2) moduli. Because operations on individual (l+2) moduli are independent of RNS decomposition, we can perform (l+2) MODs in parallel (op ②) to efficiently pipeline the computation. Modular multiplication (ModMul) also requires the original input polynomial, which reduces the number of MODs on (l+2) moduli to (l+1) MODs at a time. Figure 4 shows selectable MOD outputs. Subsequently, the (l+1) NTT modules must run in parallel for subsequent NTT computations (op ③). Once the NTT computations are complete, the ModMul module performs modular multiplications with the **SwK** using Algorithm 1. To simultaneously generate two relinearized vectors, we deployed 2 × (l+2) ModMul modules (op ④). After the ModMul product, the results were stored in the following memory banks (ops ⑤ and ⑥, respectively). We used two Ultra RAM (URAM), large-scale, high-speed memory element, banks to store two polynomials with five RNS components. After accumulating (l+1) polynomials in URAMs, the ModRai module transferred the temporary data to the ModSwi module memory and continued accumulating with the next polynomials. Cooperation after NTT was indicated as MAR, and its detailed structure is shown in Figure 5.

The ModSwi module performed the second part of the key switching operation after (l+1) iterations. In this step, temporary data from ModRai were received and stored in RAM banks (op ⑦). The following INTT unit transformed only the two polynomials with the associated special modulus $q_{sp}$ (op ⑧). The ModSwi module then performed the flooring operation with (l+1) MR units and (l+1) NTT computations (ops ⑨ and ⑩, respectively). For the ModMul operation of the 51-bit modulus, the coefficients were compared with half of $q_{sp}$, and the subtraction with the residue of $q_{sp}$ modulo $q_{i}$ was then determined [6]. At the end of the flooring, subtraction with ModRai outputs and subsequent multiplication by the inverse value of the special prime were performed for two polynomials of RNS components in parallel (ops ⑪ and ⑫, respectively). Op ⑬ added the remaining two components of the homomorphic multiplication results to the outputs of the flooring operation, and generated the relinearized ciphertext simultaneously. The output of the key switching operation consisted of two polynomials of RNS components, which are referred to as **c**${}_{0}$ and **c**${}_{1}$ of the key-switched ciphertext **c**.

**Figure 6:**
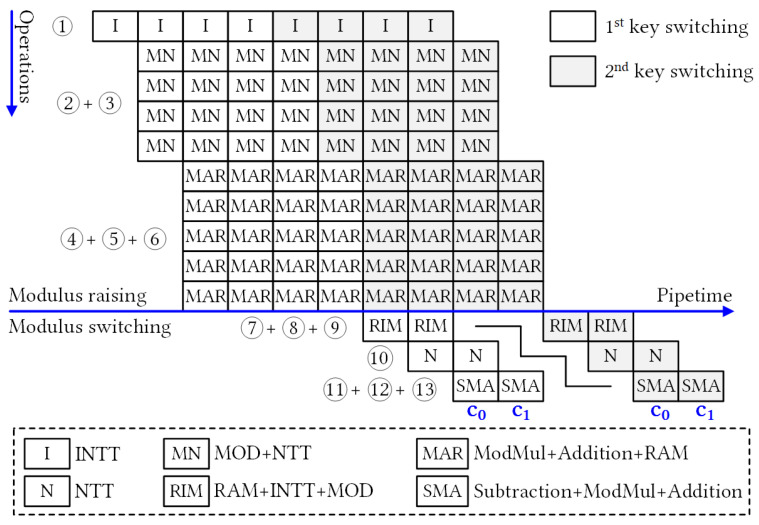
Pipelined key switching operation of consecutive ciphertext multiplications. The flow of major operations is numbered corresponding to operations in Figure 3.

The pipeline timing for the key switching operation is shown in Figure 6, where each pipeline stage comprises a series of consecutive operations separated by a few cycles. Each square block represents the approximate delay of the one-polynomial NTT computation. The ModRai unit can increase the modulus in a highly pipelined manner, with the results stored in the RAM until all input moduli are transformed (op ⑥). Subsequently, the ModSwi module performs the modulus switching operation only for two polynomials with the associated special modulus. In a pipelined operation, modulus switching has a timing delay of two square blocks. However, the delay gap between consecutive key switching operations depends on the number of modulo primes, which affects the accumulation latency in the ModRai module.

**Figure 7:**
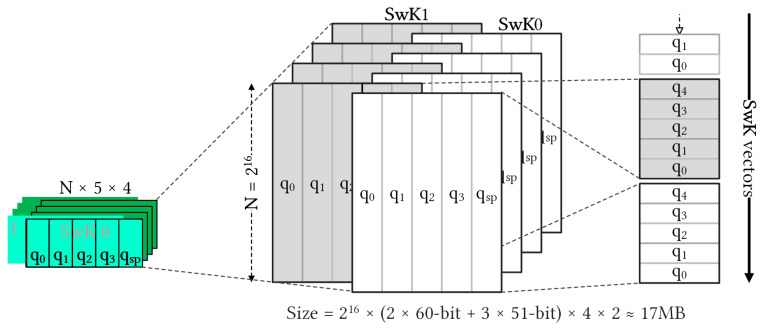
Rearrangement of **SwK** extracted from the SEAL key switching function to feed the KeySwitch module.

In this configuration of KeySwitch with l=3 and N=64 K, Figure 7 shows the tensor form of **SwK**. In the RNS domain, the component polynomials are 480 KB ($=\frac{65536\times 60\text{-}\mathrm{bit}}{1024\times 8}$) for $q_{0}$ and $q_{sp}$ with 60-bit and 408 KB ($=\frac{65536\times 51\text{-}\mathrm{bit}}{1024\times 8}$) for $q_{i}$ of 51-bit. Each ciphertext polynomial size is 1704 KB ($=\frac{65536(60\text{-}\mathrm{bit}+3\times 51\text{-}\mathrm{bit})}{1024\times 8}$), and each ciphertext size is 3408 KB. The **SwK** matrix dominated, accounting for 17,472 KB ($=4\times \frac{65536(2\times 60\text{-}\mathrm{bit}+3\times 51\text{-}\mathrm{bit})}{1024\times 8}$). The same **SwK** matrices for all homomorphic multiplication operations at a specific level can be reused. However, these matrices are often too large to be stored in the on-chip memory, leading to a significant data movement overhead and a bottleneck in the overall performance of the cryptosystem. Thus, reducing data movement between the on-chip and external memory is critical for improving the efficiency of the system.

## 5. Results and Discussion

### 5.1. Evaluation Results

We developed the proposed KeySwitch architecture using SystemVerilog HDL and converted it into register-transfer-level (RTL) designs. We then performed logic synthesis for the Xilinx UltraScale+ XCU250 FPGA platform utilizing the Xilinx Vivado (v2020.1) tool. The KeySwitch hardware design stored the TF bases in Block RAM (BRAM) units and saved temporary data in URAM. For our chosen parameter settings, we kept the **SwK** in the main memory and supplied it to the KeySwitch module for verification. With default synthesis settings, the KeySwitch module achieved a maximum clock frequency of 236 MHz.

The security level of our KeySwitch design is based directly on the CKKS FHE primitive [25], without introducing any functional modifications. Parameter choices, such as polynomial degree *N* and modulus size logQ, significantly influence the security and achievable multiplication depth of a CKKS instance. In this research, we opted for a large logQ to allow for a high circuit depth and increased *N* to ensure a higher security level. Specifically, we set $N=2^{16}$ and a large modulus of logQ=1760 bits to achieve 128-bit security [26]. These parameters allowed for a multiplication depth of up to 32 levels during ciphertext evaluation. Implementations with a circuit depth less than 32 yield a security level greater than 128 bits. We used L=3 as a study example throughout this evaluation to illustrate the effectiveness of our proposed KeySwitch module in comparison to prior work.

The synthesis results for our proposed KeySwitch module, which supports five moduli, are presented in Table 2. In the initial design, we stored all the TF constants for the utilized moduli in the on-chip BRAM. This conventional approach required a large amount of storage for precomputed TFs, leading to memory overhead. By effectively integrating TFG into the NTT and INTT hardware designs, we were able to significantly improve internal memory utilization. The NTT design approach employing runtime TFG led to a remarkable reduction (by approximately 99%) in on-chip memory usage compared to the traditional method of storing all precomputed TFs in memory. Furthermore, this approach resulted in a moderate increase (by around 21%) in DSP slices, accompanied by a negligible rise in logic elements. These outcomes highlight the effectiveness of the KeySwitch module regarding on-chip resource utilization, allowing for more internal memory allocation to evaluation keys and temporary data during calculations.

To provide a comprehensive breakdown of on-chip resource usage, Table 3 and Table 4 detail the FPGA hardware utilization of the ModRai and ModSwi modules, respectively. The functional modules corresponding to the operations shown in Figure 3 were synthesized and reported separately. This approach facilitates a more precise assessment of resource utilization. With the NTT and INTT modules operating on a single modulus, we were able to derive the TF memory from LUTRAM instead of BRAM, resulting in significant savings in on-chip RAM utilization. Additionally, it is worth noting that 60-bit integer multiplier necessitated the use of twelve DSP slices, while 51-bit integer multiplier only necessitated six DSP slices. As a result, we developed various NTT modules for different moduli to maximize the utilization of DSP slices.

Table 3 shows that the ModRai module dominates on-chip resource consumption in the KeySwitch hardware design. In particular, the INTT unit consumed 12.5 BRAMs to store the TF bases of four moduli. The moduli switching circuit used more LUT elements and FFs. The first three NTT units alternatively operated on two modulo primes and shared the multiplexing circuit from the previous MOD units to select the appropriate modulus. The associated RTL designs of these NTT units are denoted as NTT$q_{0123}$, in which NTT$q_{01}$, NTT$q_{12}$, and NTT$q_{23}$ consume 564, 282, and 282 DSP slices and 12.5, 11, and 11 BRAMs, respectively. For dyadic multiplication and accumulation, we grouped the RTL modules into designs denoted as MARs of the corresponding modulus primes. Each unit simultaneously processed 16 coefficients during key switching.

Table 4 provides a clear breakdown of the hardware consumption of subunits in the ModSwi module. The INTT and NTT units in this module operate only on a singular modulus, which is the reason we derived the TF memories from LUTRAM. To simplify the design, we grouped the RTL modules of the four NTT units into a single design, denoted as NTT$q_{0123}$, because they shared the same control circuit. The DSP utilization of the NTT unit of $q_{0}$ was 564 DSP slices, whereas each NTT unit of the others $q_{i}$ consumed only 282 slices. For the KeySwitch module, we utilized URAM to construct temporary data memory units. Using our design method, we confirmed that the memory unit of each RNS component consistently consumed 16 URAM blocks for the 16-bank data memory units.

### 5.2. Comparison with Related Works

Comparing with the software implementation, we used a computer system equipped with an Intel Core i9-9900KF CPU, 32 GB DDR4 DRAM that runs on the Windows 10 operating system. We installed version 3.7 of the widely used SEAL HE library [6] and then executed the switch_key_inplace() routine to evaluate the execution time of key switching. Latency measurements were performed using Chrono C++ functions. Then, we extracted the test vectors from the SEAL source code and ran them through the KeySwitch module for verification. As shown in Table 5, our KeySwitch design achieved a speedup of approximately 113.4× compared with the software implementation.

The most suitable comparison for our key switching accelerator is with HEAX [14]. In Table 6, we compare the efficiency of both KeySwitch hardware designs. Even though the polynomial sizes differ, both studies performed key switching at the same circuit depth, enabling fair comparisons. We calculated data throughput and assessed hardware efficiency metrics for this comparison. Our KeySwitch module design operates at a lower clock frequency on Xilinx FPGA technology than HEAX but achieves a 1.6× higher data throughput. Comparing LUT efficiencies is impractical due to structural differences between Intel FPGA’s ALM elements and Xilinx FPGA’s LUT elements. Differences in DSP slice structures between the two FPGA technologies led to distinct modulus bit width selections. Although our design exhibited lower DSP efficiency, we employed enhanced Barrett-based modular multiplication and reasonable numbers of DSP slices, combined with lightweight modular reduction. Our KeySwitch design also used flip-flops more effectively than HEAX for pipelined registers. Importantly, our proposed KeySwitch design achieved a 2.15× improvement in RAM efficiency. Despite a 10× larger polynomial size, our KeySwitch module consumed 1.3× less internal RAM than HEAX. The primary advantage of our design lies in the use of TFG modules in the NTT and INTT hardware designs, as well as the minimal number of TF constants stored in on-chip memory.

Comparing with other FPGA-based implementations: Medha presents a single hardware design for RNS-CKKS acceleration using a Xilinx Alveo U250 FPGA, offering a versatile instruction-set architecture that supports two HE parameter sets (Set-1: $N=2^{14},\log Q=438$ bits and Set-2: $N=2^{15},\log Q=564$ bits) [17]. With a 497.24 μs execution time of homomorphic multiplication for Set-1, Medha reaches a throughput rate of 14,431 Mbps. In contrast, our design employs a pipelined strategy, achieving 3.4× higher throughput than Medha at the cost of increased hardware resource usage. Poseidon, an FPGA-based FHE accelerator featuring bootstrapping capabilities, utilizes optimization methods to enhance resource efficiency [18]. By leveraging an advanced Xilinx Alveo U280 FPGA with high-bandwidth memory (HBM), Poseidon reports a key switching latency of 218.6 μs for a specific parameter set of ($N=2^{16},L=44$). Our KeySwitch module exhibits a comparable execution time of 284.6 μs, but with reduced hardware overhead. FAB, an additional U280 FPGA-based FHE accelerator with bootstrapping support, refines on-chip memory access to remove memory-access-related bottlenecks [19]. For a parameter set of ($N=2^{14},\log Q=438$ bits), FAB attains an execution time of 180.3 μs for homomorphic multiplication and a throughput rate of 39,802 Mbps, which is marginally lower than our KeySwitch module’s 49,046 Mbps. Nonetheless, FAB consumes a higher hardware ratio than our design, with the exception of DSP slices. To summarize, our design focuses on accelerating key switching using pipelined and parallel implementations. By deploying the processor in consecutive pipeline stages, key switching operations are unrolled, resulting in high asymptotic throughput with minimal hardware resource overhead.

Comparing with 100×, the GPU-based FHE implementation by Jung et al. [8]: The 100× focuses on large parameter sets ($N=2^{16},\log Q=2364$ bits and $N=2^{17},\log Q=3220$ bits) and achieves a significant speedup for CKKS compared to previous GPU-based attempts. Through memory-centric improvements, 100× enhances overall performance and reaches an acceleration rate more than 100 times faster than single-threaded CPU execution. While it is challenging to make a fair comparison between their work and our architecture, our KeySwitch module attains a similar processing time with a more adaptable and customizable FPGA technology implementation. In addition, there are some other studies that demonstrate impressive performance by utilizing modern GPU features, such as tensor cores. For instance, TensorFHE accelerates NTT computation by adopting GPU fine-grained operation and data parallelism [9]. However, TensorFHE still faces suboptimal acceleration due to GPU architectural limitations.

### 5.3. Limitation of This Study

As shown in Figure 7, **SwK** is larger than the on-chip BRAM capacity. Although **SwK** is reusable, the internal memory of existing FPGA devices for storing all **SwK** remains an overhead. To mitigate this issue, we reserved on-chip BRAMs for intermediate data and stored **SwK** test vectors in the main memory. However, this resulted in data movement from the main memory becoming a critical performance bottleneck, thereby limiting the acceleration of the KeySwitch module. An effective solution to further increase the main memory bandwidth is to use alternative main memory technologies, such as HBM [27]. HBM can provide several times higher bandwidth than the DDRx technology, thus improving the performance of the KeySwitch module.

Han and Ki proposed a method to reduce the length of **SwK** by using a decomposition number (dnum) to split **SwK** and decompose ciphertexts into dnum slices [26]. However, an increasing dnum also increases the number of **SwK** components. To overcome this limitation, we can store each component at each computation time, which reduces the number of accesses to the external memory during key switching. Choosing a proper dnum is crucial to strike a balance between the multiplication depth and homomorphic evaluation complexity. Furthermore, the NTT and INTT units perform computations iteratively, and the **SwK** components are cached in the internal buffer over time. Therefore, the use of dnum can significantly reduce the **SwK** length, whereas careful consideration of the trade-offs can enhance the overall performance of the KeySwitch module.

## 6. Conclusions

This study proposed an efficient hardware design for the KeySwitch module that accelerates the homomorphic multiplication by utilizing efficient NTT and INTT engines. The KeySwitch module achieved high hardware efficiency by utilizing on-chip resources and reducing the internal memory consumption. The pipelined key switching operation also enabled fast homomorphic multiplication with high-throughput rates.

In the future, the proposed KeySwitch module can be applied to accelerate realistic HE-based applications, such as logistic regression inference and simple convolutional neural networks. Efficient NTT and INTT hardware designs can support large circuit depths, making the instruction-set KeySwitch architecture a promising approach for practical HE-based applications. Further research should investigate the integration of the proposed KeySwitch module with other HE-based cryptographic schemes to develop a more comprehensive hardware acceleration platform.

## Figures and Tables

**Table 1:** Overview of CKKS-supported HE accelerations.

Name	Year	Hardware	Design	N	L${}_{\max}$	Acceleration
Privft [7]	2020	GPU	SW	$2^{10}$–$2^{16}$	44	Leveled HE
100x [8]	2021	GPU	SW	$2^{16}$–$2^{17}$	44	Bootstrapping
TensorFHE [9]	2023	GPU	SW	$2^{15}$–$2^{16}$	57	Bootstrapping
F1 [10]	2021	ASIC	SW/HW	$2^{12}$–$2^{14}$	24	Bootstrapping
CraterLake [11]	2022	ASIC	SW/HW	$2^{11}$–$2^{16}$	60	Bootstrapping
BTS [12]	2022	ASIC	SW/HW	$2^{17}$	44	Bootstrapping
ARK [13]	2022	ASIC	SW/HW	$2^{16}$	23	Bootstrapping
HEAX [14]	2020	FPGA	RTL	$2^{12}$–$2^{14}$	7	Leveled HE
HEXL-FPGA [15]	2021	FPGA	HLS	$2^{10}$–$2^{14}$	7	Leveled HE
coxHE [16]	2022	FPGA	HLS	$2^{11}$–$2^{13}$	3	Leveled HE
Medha [17]	2023	FPGA	RTL	$2^{14}$–$2^{15}$	9	Leveled HE
Poseidon [18]	2023	FPGA	HLS	$2^{16}$	57	Bootstrapping
FAB [19]	2023	FPGA	RTL	$2^{16}$	23	Bootstrapping

**Table 2:** Hardware consumption of the KeySwitch module on the Xilinx XCU250 FPGA platform.

Design		LUT	FF	DSP	BRAM *	URAM *
		(%)	(%)	(%)	(%)	(%)
KeySwitch module	w/o TFG	816,188	796,331	5376	1842	464
		(47%)	(23%)	(44%)	(69%)	(36%)
	w/TFG	850,843	887,095	6534	47	464
		(49%)	(26%)	(53%)	(2%)	(36%)
Xilinx XCU250		1,728,000	3,456,000	12,288	2688	1280

* This study explicitly employed BRAM to store TF constants and URAM to store polynomial coefficients.

**Table 3:** FPGA resource breakdown of the ModRai module.

Ops	Module	LUT	FF	DSP	BRAM	URAM	No. ${}^{1}$
①	INTT	105,563	86,401	564	12.5	16	1
②	MOD	14,496	14,720	0	0	0	4
③	NTT$q_{0123}$ ${}^{3}$	187,974	160,101	1128	34.5	48	3
	NTT$q_{sp}$	54,294	56,073	564	0 ${}^{2}$	16	1
④	MAR$q_{0}$	27,904	41,216	384	0	32	2
⑤	MAR$q_{123}$	77,376	97,056	576	0	96	6
⑥	MAR$q_{sp}$	22,912	37,408	384	0	32	2
	ModRai	490,519	492,975	3600	47.0	240	

${}^{1}$ No. denotes the number of separate units in each corresponding Ops. ${}^{2}$ TF memory for NTT module of only one modulus is derived from LUTRAM. ${}^{3}$ NTT$q_{0123}$ consists of three NTT modules for respective $q_{01}$, $q_{12}$, and $q_{23}$. Because of different modulus sizes, NTT$q_{01}$ module consumes 564 DSP slices while $q_{12}$ and $q_{23}$ consume 282 DSP slices each.

**Table 4:** FPGA resource breakdown of the ModSwi module.

Ops	Module	LUT	FF	DSP	BRAM	URAM	No. ${}^{1}$
⑦	RAM	25	25	0	0	144	9
⑧	INTT	53,758	56,016	564	0 ${}^{2}$	16	1
⑨	MOD	20,368	15,440	0	0	0	4
⑩	NTT$q_{0123}$	182,977	193,527	1410	0 ${}^{2}$	64	4
⑪	SM$q_{0}$	21,120	33,248	384	0	0	2
⑫	SM$q_{123}$	64,992	79,104	576	0	0	6
⑬	ADD	17,083	16,760	0	0	0	8
	ModSwi	360,324	394,120	2934	0	224	

${}^{1}$ No. denotes the number of separate units in each corresponding Ops. ${}^{2}$ TF memory for NTT and INTT modules of only one modulus are derived from LUTRAM.

**Table 5:** Comparison with software implementation on SEAL HE library [6].

Parametrics	Key Switching	
Device	CPU (SEAL)	Xilinx Alveo U250
No. of CCs	-	67,168
Clock frequency	3.6 GHz	236 MHz
Latency (μs)	32,402	284.6
No. of ops/s	31	3514 (113.4×)

**Table 6:** Comparison of the KeySwitch module with the most related work.

Parametrics	HEAX [14]	This Work
Device	Intel Stratix10	Xilinx Alveo U250
*N*	$2^{13}$	$2^{16}$
Max. prime (bits)	54	60
logQ (bits)	218	273
Levels	3	3
Frequency (MHz)	300	236
ALM/LUT	699 K (ALM) ${}^{*}$	851 K (LUT)
REG/FF	1976 K (REG)	887 K (FF)
DSP slices	2610	6534
RAM (MB)	22	16.5
Throughput (Mbps)	30,279	49,046
Norm. Throu./REG	1	3.6
Norm. Throu./DSP	1	0.65
Norm. Throu./RAM	1	2.15

${}^{*}$ ALM (adaptive logic module) is a core logic unit including two combinational adaptive LUTs, a two-bit full adder, and four 1-bit REGs.

## Data Availability

Not applicable.

## References

[1] Rivest R.L., Adleman L., Dertouzos M.L. (1978). On data bank and privacy homomorphisms. Found. Secur. Comput..

[2] Gentry C. Fully Homomorphic Encryption Using Ideal Lattices. Proceedings of the Forty-First Annual ACM Symposium on Theory of Computing.

[3] Regev O. (2009). On lattices, learning with errors, random linear codes, and cryptography. J. ACM.

[4] Cheon J.H., Costache A., Moreno R.C., Dai W., Gama N., Georgieva M., Halevi S., Kim M., Kim S., Laine K., Lauter K., Dai W., Laine K. (2022). Introduction to Homomorphic Encryption and Schemes. Protecting Privacy through Homomorphic Encryption.

[5] Albrecht M., Chase M., Chen H., Ding J., Goldwasser S., Gorbunov S., Halevi S., Hoffstein J., Laine K., Lauter K., Lauter K., Dai W., Laine K. (2022). Homomorphic encryption standards. Protecting Privacy through Homomorphic Encryption.

[6] Microsoft SEAL (Release 3.7) Microsoft Research.

[7] Badawi A.A., Hoang L., Mun C.F., Laine K., Aung K.M. (2020). Privft: Private and fast text classification with homomorphic encryption. IEEE Access.

[8] Jung W., Kim S., Ahn J.H., Cheon J.H., Lee Y. (2021). Over 100x faster bootstrapping in fully homomorphic encryption through memory-centric optimization with gpus. IACR Trans. Cryptogr. Hardw. Embed. Syst..

[9] Fan S., Wang Z., Xu W., Hou R., Meng D., Zhang M. Tensorfhe: Achieving practical computation on encrypted data using gpgpu. Proceedings of the IEEE International Symposium on High-Performance Computer Architecture (HPCA).

[10] Samardzic N., Feldmann A., Krastev A., Devadas S., Dreslinski R., Peikert C., Sanchez D. F1: A Fast and Programmable Accelerator for Fully Homomorphic Encryption. Proceedings of the Twenty-Fifth International Conference on Architectural Support for Programming Languages and Operating Systems (MICRO).

[11] Samardzic N., Feldmann A., Krastev A., Manohar N., Genise N., Devadas S., Eldefrawy K., Dreslinski R., Peikert C., Sanchez D. Craterlake: A hardware accelerator for efficient unbounded computation on encrypted data. Proceedings of the 49th Annual International Symposium on Computer Architecture (ISCA).

[12] Kim S., Kim J., Kim M.J., Jung W., Kim J., Rhu M., Ahn J.H. BTS: An accelerator for bootstrappable fully homomorphic encryption. Proceedings of the 49th Annual International Symposium on Computer Architecture (ISCA).

[13] Kim J., Lee G., Kim S., Sohn G., Rhu M., Kim J., Ahn J.H. ARK: Fully homomorphic encryption accelerator with runtime data generation and inter-operation key reuse. Proceedings of the 55th IEEE/ACM International Symposium on Microarchitecture (MICRO).

[14] Riazi M.S., Laine K., Pelton B., Dai W. HEAX: Architecture for computing encrypted data. Proceedings of the Twenty-Fifth International Conference on Architectural Support for Programming Languages and Operating Systems.

[15] Meng Y., Butt S., Wang Y., Zhou Y., Simoni S., Abu-Alam P., Aragon T.G., Bergamaschi F., de Lassus H., de Souza F.D.M. (2022). Intel Homomorphic Encryption Acceleration Library for FPGAs (Version 2.0). https://github.com/intel/hexl-fpga.

[16] Han M., Zhu Y., Lou Q., Zhou Z., Guo S., Ju L. coxHE: A software-hardware co-design framework for FPGA acceleration of homomorphic computation. Proceedings of the Design, Automation & Test in Europe Conference & Exhibition (DATE).

[17] Mert A.C., Kwon S., Shin Y., Yoo D., Lee Y., Roy S.S. (2023). Medha: Microcoded hardware accelerator for computing on encrypted data. IACR Trans. Cryptogr. Hardw. Embed. Syst..

[18] Yang Y., Zhang H., Fan S., Lu H., Zhang M., Li X. Poseidon: Practical Homomorphic Encryption Accelerator. Proceedings of the IEEE International Symposium on High-Performance Computer Architecture (HPCA).

[19] Agrawal R., de Castro L., Yang G., Juvekar C., Yazicigil R., Chandrakasan A., Vaikuntanathan V., Joshi A. FAB: An FPGA-based accelerator for bootstrappable fully homomorphic encryption. Proceedings of the IEEE International Symposium on High-Performance Computer Architecture (HPCA).

[20] Duong-Ngoc P., Kwon S., Yoo D., Lee H. (2023). Area-efficient number-theoretical transform architecture for Homomorphic encryption. IEEE Trans. Circuits Syst. I Regul. Pap..

[21] Crandall R., Pomerance C. (2005). Prime Numbers: A Computational Perspective.

[22] Micciancio D., Peikert C. Trapdoors for Lattices: Simpler, Tighter, Faster, and Smaller. Proceedings of the 31st Annual International Conference on the Theory and Applications of Cryptographic Techniques.

[23] Chen H., Dai W., Kim M., Song Y., Sako K., Tippenhauer N.O. (2021). Efficient homomorphic conversion between (ring) LWE ciphertexts. Applied Cryptography and Network Security (ACNS 2021), Lunk Notes in Computer Science (LNCS).

[24] Cheon J.H., Han K., Kim A., Kim M., Song Y., Cid C., Jacobson M. (2018). Full RNS variant of the approximate homomorphic encryption. Selected Areas in Cryptography (SAC, 2018), Lunk Notes in Computer Science (LNCS).

[25] Cheon J.H., Kim A., Kim M., Song Y. Homomorphic Encryption for Arithmetic of Approximate Numbers. Proceedings of the Advances in Cryptology–ASIACRYPT 2017:23rd International Conference on the Theory and Applications of Cryptology and Information Security.

[26] Han K., Ki D., Jarecki S. (2020). Better Bootstrapping for approximate homomorphic Encryption. Topics in Cryptology (CT-RSA 2020), Lecture Notes in Computer Science (LNCS).

[27] Jun H., Cho J., Lee K., Son H.Y., Kim K., Jin H., Kim K. HBM (High Bandwidth Memory (HBM) Drama Technology and Architecture. Proceedings of the IEEE International Memory Workshop (IMW).

